# β5 Integrin Up-Regulation in Brain-Derived Neurotrophic Factor Promotes Cell Motility in Human Chondrosarcoma

**DOI:** 10.1371/journal.pone.0067990

**Published:** 2013-07-09

**Authors:** Chih-Yang Lin, Hui-Jye Chen, Te-Mao Li, Yi-Chin Fong, Shan-Chi Liu, Po-Chun Chen, Chih-Hsin Tang

**Affiliations:** 1 Graduate Institute of Basic Medical Science, China Medical University, Taichung, Taiwan; 2 Graduate Institute of Molecular Systems Biomedicine, China Medical University, Taichung, Taiwan; 3 School of Chinese Medicine, China Medical University, Taichung, Taiwan; 4 Department of Orthopaedics, China Medical University Hospital, Taichung, Taiwan; 5 Institute of Biomedical Sciences, National Chung Hsing University, Taichung, Taiwan; 6 Department of Life Sciences, National Chung Hsing University, Taichung, Taiwan; 7 Department of Pharmacology, School of Medicine, China Medical University, Taichung, Taiwan; 8 Department of Biotechnology, College of Health Science, Asia University, Taichung, Taiwan; University of Bergen, Norway

## Abstract

Chondrosarcoma is a primary malignant bone cancer, with a potent capacity to invade locally and cause distant metastasis; it has a poor prognosis and shows a predilection for metastasis to the lungs. Brain derived neurotrophic factor (BDNF) is a small-molecule protein from the neurotrophin family of growth factors that is associated with the disease status and outcomes of cancers. However, the effect of BDNF on migration activity in human chondrosarcoma cells is mostly unknown. Here, we found that human chondrosarcoma tissues showed significant expression of BDNF, which was higher than that in normal cartilage and primary chondrocytes. We also found that BDNF increased the migration and expression of β5 integrin in human chondrosarcoma cells. In addition, knockdown of BDNF expression markedly inhibited migratory activity. BDNF-mediated migration and β5 integrin up-regulation were attenuated by antibody, inhibitor, or siRNA against the TrkB receptor. Pretreatment of chondrosarcoma cells with PI3K, Akt, and NF-κB inhibitors or mutants also abolished BDNF-promoted migration and integrin expression. The PI3K, Akt, and NF-κB signaling pathway was activated after BDNF treatment. Taken together, our results indicate that BDNF enhances the migration of chondrosarcoma by increasing β5 integrin expression through a signal transduction pathway that involves the TrkB receptor, PI3K, Akt, and NF-κB. BDNF thus represents a promising new target for treating chondrosarcoma metastasis.

## Introduction

Brain-derived neurotrophic factor (BDNF) is a small basic protein that is highly conserved among different species. In addition, BDNF is widely distributed in various types of tissues [1], [2], [3], [4]. BDNF and its receptor TrkB play key roles in neural development, and some studies have suggested a role for BDNF in cancer cell proliferation, survival, differentiation, and invasiveness [5], [6]. For example, BDNF protects neuroblastoma cells from chemotherapeutic agent induced cytotoxicity [7].

Chondrosarcomas are a heterogeneous group of neoplasms that share in common the production of cartilage matrix by the tumor cells. It is an uncommon, malignant primary bone tumor with a poor prognosis that may occur at any age between 10 and 80 years. Approximately two-thirds of the affected patients are male [8], and the tumor usually appears on scapula, sternum, ribs, or pelvis [9]. Clinically, surgical resection remains the primary mode of therapy for chondrosarcoma. Due to the absence of an effective adjuvant therapy, this mesenchymal malignancy has a poor prognosis and, therefore, it is important to explore novel remedies [10].

Tumor invasion and metastasis are the main biological characteristics of cancer cells [11]. Mortality in cancer patients principally results from metastatic spread of cancer cells to distant organs. Tumor metastasis is a highly complex multistep process, which includes changes in cell-cell adhesion properties [11]. Because integrins expressed on the surface of a cell determine whether the cell can adhere to and survive in a particular microenvironment, the matching of integrins and ligands plays a key role [12]. Integrins are a family of transmembrane glycoprotein adhesion receptors that play central roles in the biology of metazoans by controlling cell adhesion, migration, differentiation, and apoptosis. Integrins form heterodimers of α and β subunits [13]. There are at least 19 α subunits and 8 β subunits that can associate to form 25 unique integrin heterodimers [14], [15]. Integrins play an important role in many extracellular matrix (ECM) matrix proteins such as collagens, fibronectin, laminin, osteopontin, and vitronectin [16]. In addition, integrins have also been implicated in metastasis of lung, breast, bladder, colon cancer, and chondrosarcomas [17], [18], [19], [20].

Previous studies have shown that BDNF increases cell migration and invasion in human cancer cells [21], [22]. However, the effect of BDNF on integrin expression and migration activity in human chondrosarcoma cells is not well understood. We therefore examined whether BDNF promoted integrin expression and cell motility in human chondrosarcoma cells. Here, we found that BDNF increases migration and up-regulates β5 integrin in human chondrosarcoma cells. Moreover, the TrkB receptor, phosphatidylinositol 3′-kinase (PI3K), Akt, and NF-κB signaling pathways were shown to be involved.

## Materials and Methods

### Materials

Anti-rabbit and anti-mouse IgG-conjugated horseradish peroxidase, mouse monoclonal antibody specific for β5 integrin was purchased from Chemicon (Temecula, CA). Rabbit polyclonal antibodies specific for BDNF, TrkB, p-p85, Akt, p-Akt, p65, IKKα/β, p-IKKα/β, and IκB, and were purchased from Santa Cruz Biotechnology (Santa Cruz, CA). TPCK and pyrrolidine dithiocarbamate (PDTC) were purchased from Calbiochem (San Diego, CA). Recombinant human BDNF was purchased from R&D Systems (Minneapolis, MN, USA). NF-κB luciferase plasmid was purchased from Stratagene (La Jolla, CA). The p85 and Akt (Akt K179A) dominant-negative mutants were gifts from Dr. W.M. Fu (National Taiwan University, Taipei, Taiwan). IKKα (KM) and IKKβ (KM) mutants were gifts from Dr. H. Nakano (Juntendo University, Tokyo, Japan). The pSV-β-galactosidase vector and the luciferase assay kit were purchased from Promega (Madison, MA). All other chemicals were purchased from Sigma-Aldrich (St. Louis, MO).

### Patients and Specimen Preparation

The study protocol was approved by the Institutional Review Board of China Medical University Hospital, and all subjects gave informed written consent before enrollment. The specimens of tumor tissue or normal cartilage tissue were obtained from patients who were had been diagnosed with chondrosarcoma or knee osteoarthritis and had undergone surgical resection at China Medical University Hospital. Tissue specimens were ground and then sonicated in protein lysis buffer. Protein levels were analyzed by western blotting.

### Immunohistochemistry

Paraffin-embedded tissues were cut into 5-µm-thick sections and mounted on glass slides. After rehydration and incubation in 3% hydrogen peroxide to block endogenous peroxidase activity, sections were blocked by incubation in 3% bovine serum albumin in PBS. The monoclonal mouse anti-human BDNF primary antibody was applied to the slides at a dilution of 1∶50 and incubated at 4°C overnight. After 3 washes in PBS, the samples were treated with goat anti-mouse IgG biotin-labeled secondary antibodies at a dilution of 1∶50. Bound antibodies were detected with an ABC kit (Vector Laboratories). The slides were stained with a chromogen diaminobenzidine, washed, counterstained with Delafield’s hematoxylin, dehydrated, treated with xylene, and then mounted. The staining intensity was scored from 1 to 5.

### Cell Culture

The human chondrosarcoma cell line (JJ012) was kindly provided by Dr. Sean P. Scully’s laboratory (University of Miami School of Medicine, Miami, FL, USA) [23]. Cells were cultured in Dulbecco’s modified Eagle’s medium (DMEM)/α-MEM supplemented with 10% fetal bovine serum (FBS). The human chondrosarcoma cell line (SW1353) was obtained from the American Type Culture Collection. The cells were cultured in DMEM supplemented with 10% FBS. These cells were maintained at 37°C in a humidified atmosphere of 5% CO_2_.

To establish primary cultures, chondrocytes were isolated from articular cartilage as previously described [24]. The cells were grown in plastic cell culture dishes in 95% air-5% CO_2_ in DMEM supplied with 20 mM HEPES, 10% heat-inactivated FBS, 2 mM-glutamine, 100 U/ml penicillin, and 100 µg/ml streptomycin.

### Migration Assay

Migration activity was measured using a Transwell assay (Costar, NY; pore size, 8-µm) and Transwell membrane was pre-coated with β5 integrin ligand vitronectin (30 µl, 1 µg/ml). For the invasion assay, filters were precoated with 30 µl Matrigel basement membrane matrix (BD Biosciences, Bedford, MA) for 30 min. The following procedures were conducted in the same manner for both the migration and invasion assays. Before the migration assay was performed, cells were pretreated for 30 min with different concentrations of inhibitors, including the K252a, Ly294002, wortmannin, Akt inhibitor, or vehicle control (0.1% dimethyl sulfoxide). Approximately 1.5×10^4^ cells were added to the upper chamber in 200 µl of serum-free medium. The lower chamber in 300 µl of serum-free medium was applied with BDNF. The plates were incubated for 24 h at 37°C in 5% CO_2_, and then cells were fixed in 3.7% formaldehyde solution for 15 min and stained with 0.05% crystal violet in PBS for 15 min. Cells on the upper side of the filters were removed with cotton-tipped swabs, and the filters were washed with PBS. On the other hand, cells on the underside of the filters were examined and counted under a microscope. Each clone was plated in triplicate for each experiment, and each experiment was repeated at least 3 times. The number of migrating cells in each experiment was adjusted according to the results of the cell viability assay to correct for proliferation effects of the BDNF treatment (corrected migrating cell number = counted migrating cell number/percentage of viable cells) [25].

### Wound-healing Migration Assay

For the wound-healing migration assay, cells were seeded on 12-well plates at a density of 1×10^5^ cells/well in culture medium. At 24 h after seeding, the monolayer cells were manually scratched with a pipette blue tip to create extended and definite scratches in the center of the dishes with a bright and clear field (∼2 mm). The detached cells were removed by washing the cells once with PBS. Serum-free medium with or without BDNF was added to each dish. After 24 h migrated, the numbers of migratory cells were counted from the resulting three images for each point and then averaged for each experimental condition. The results were compared with control group after 24 h migrated.

### Quantitative Real-time Polymerase Chain Reaction

Total RNA was extracted from chondrosarcoma cells using a TRIzol kit (MDBio Inc., Taipei, Taiwan). The reverse transcription reaction was performed using 1 µg of total RNA that was reverse transcribed into cDNA using oligo(dT) primers [26]. Quantitative real-time PCR (qPCR) analysis was carried out using Taqman® One-Step RT-PCR Master Mix (Applied Biosystems, CA). Two microliters of cDNA template was added to each 25-µl reaction with sequence-specific primers and Taqman® probes. Sequences for all target gene primers and probes were purchased commercially. Glyceraldehyde 3-phosphate dehydrogenase (GAPDH) was used as an endogenous control to normalize expression data (Applied Biosystems). qPCR assays were carried out in triplicate on a StepOnePlus sequence detection system. The cycling conditions were as follows: initial 10-min polymerase activation at 95°C followed by 40 cycles at 95°C for 15 s and 60°C for 60 s. To calculate the cycle number at which the transcript was detected (CT), the threshold was set above the non-template control background and within the linear phase of target gene amplification.

### Western Blot Analysis

Cellular lysates were prepared as described previously [27]. Proteins were resolved on sodium dodecyl sulfate-polyacrylamide gel electrophoresis and transferred to Immobilon polyvinyl difluoride membranes. The blots were blocked with 4% nonfat milk for 1 h at room temperature and then probed with rabbit anti-human antibodies against p85, p-p85, Akt, p-Akt, IKKα/β, p-IKKα/β, IκB, p-IκB, p65, p-p65, BDNF, and TrkB (1∶1000) for 1 h at room temperature (Santa Cruz, CA). After 3 washes, the blots were subsequently incubated with a goat anti-rabbit or goat anti-mouse peroxidase-conjugated secondary antibody (1∶1000) for 1 h at room temperature. The blots were visualized by enhanced chemiluminescence using Kodak X-OMAT LS film (Eastman Kodak, Rochester, NY).

### Flow Cytometric Analysis

Human chondrosarcoma cells were plated in 6-well dishes. The cells were then washed with PBS, detached with trypsin at 37°C, and fixed in 75% ethanol overnight. After being rinsed in PBS, the cells were incubated with mouse anti-human antibody against integrin β5 [(AST-3T, BioLegend); 1∶100] for 1 h at room temperature. Subsequently, cells were washed again with PBS and incubated with fluorescein isothiocyanate (FITC)-conjugated goat anti-mouse secondary IgG (1∶100; Leinco Technologies Inc., St. Louis, MO, USA) for 60 min in the dark. The labeled cells were rinsed in wash buffer, and analyzed by flow cytometry using FACS Calibur and CellQuest software (BD Biosciences). (Figure S1).

### Transient Transfection and Reporter Gene Assay

Human chondrosarcoma cells were plated in 12-well dishes, and cells were grown to 80% confluence. DNA and Lipofectamine 2000 (LF2000; Invitrogen) were premixed for 20 min and then applied to the cells. Twenty-four hours after transfection, the cells were incubated with the indicated agents. Following a further 24 h of incubation, the media were removed, and cells were washed once with cold PBS. To prepare lysates, 100 µl reporter lysis buffer (Promega, Madison, WI) was added to each well, and cells were scraped from dishes. The supernatant was collected after centrifugation at 13,000 rpm for 5 min. Aliquots of cell lysate (20 µl) containing equal amounts of protein (20–30 µg) were placed in the wells of an opaque black 96-well microplate. An equal volume of luciferase substrate was added to all samples, and luminescence was measured using a microplate luminometer. The value for luciferase activity was normalized to transfection efficiency monitored by the co-transfected β-galactosidase expression vector.

### Immunofluorocytochemistry

Cells were cultured in 12-mm coverslips. After treatment with BDNF, cells were fixed with 4% paraformaldehyde at room temperature. Thirty minutes later, 0.5% Triton X-100 was added to the cells. The cells were then incubated with rabbit anti-p65 (1∶100) and FITC-conjugated goat anti-rabbit secondary antibody (1∶100; Leinco Technologies Inc) for 1 h. Finally, cells stained with DAPI for 5 min were washed, mounted, and examined with a Zeiss fluorescence microscope.

### Establishment of Stably Transfected Cells

Cells were transfected with BDNF shRNA or control shRNA plasmids with the Lipofectamine 2000 transfection reagent (Invitrogen, Carlsbad, CA). After 24 h, cells were trypsinized and replated in DMEM/α-MEM supplemented with 10% FBS and maintained at 37°C in a humidified atmosphere of 5% CO_2_. Subsequently, 10 µg/mL puromycin (Life Technologies) was used to select stable transfectants. Thereafter, the selection medium was replaced every 3 days. After 2 weeks of selection in puromycin, clones of resistant cells were isolated.

### Statistical Analysis

Data are presented as mean ± standard error of the mean. Statistical analysis of comparisons between 2 samples was performed using the Student’s *t* test. Statistical comparisons of more than 2 groups were performed using one-way analysis of variance with Bonferroni’s post-hoc test. In all cases, *p*<0.05 was considered significant.

## Results

### BDNF Increases Cell Motility in Human Chondrosarcoma Cells

**Figure 1:**
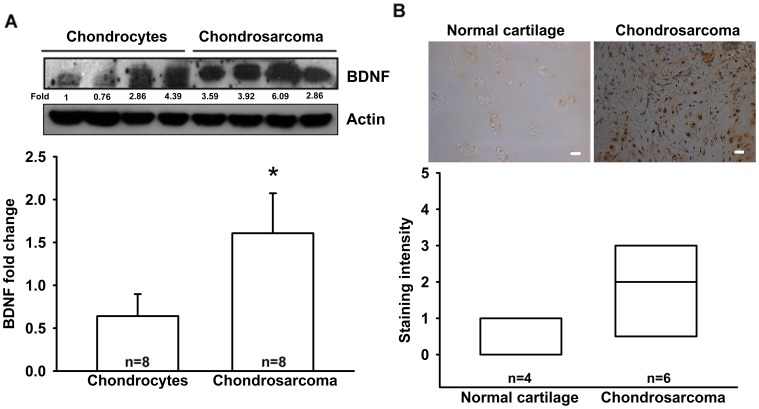
BDNF expressed in human chondrosarcoma patients. (A) Total proteins were extracted from chondrosarcoma patients (n = 8) and primary chondrocytes (n = 8), and subjected to western blot analysis for BDNF. (B) Immunohistochemistry of BDNF expression in normal cartilage (n = 4) and chondrosarcoma tissue (n = 6). Size bar = 20 µm. Results are expressed as the mean ±standard error of mean (SEM).

BDNF has been reported to be expressed by many types of human cancer cells [28], [29]. However, little is known about the expression of BDNF in human chondrosarcoma cells. We therefore examined the expression of BDNF in human chondrosarcoma patients by using immunohistochemistry and western blotting. We found that protein expression levels of BDNF in chondrosarcoma patients were significantly higher than in primary chondrocytes and normal cartilage (Fig. 1A&B).

**Figure 2:**
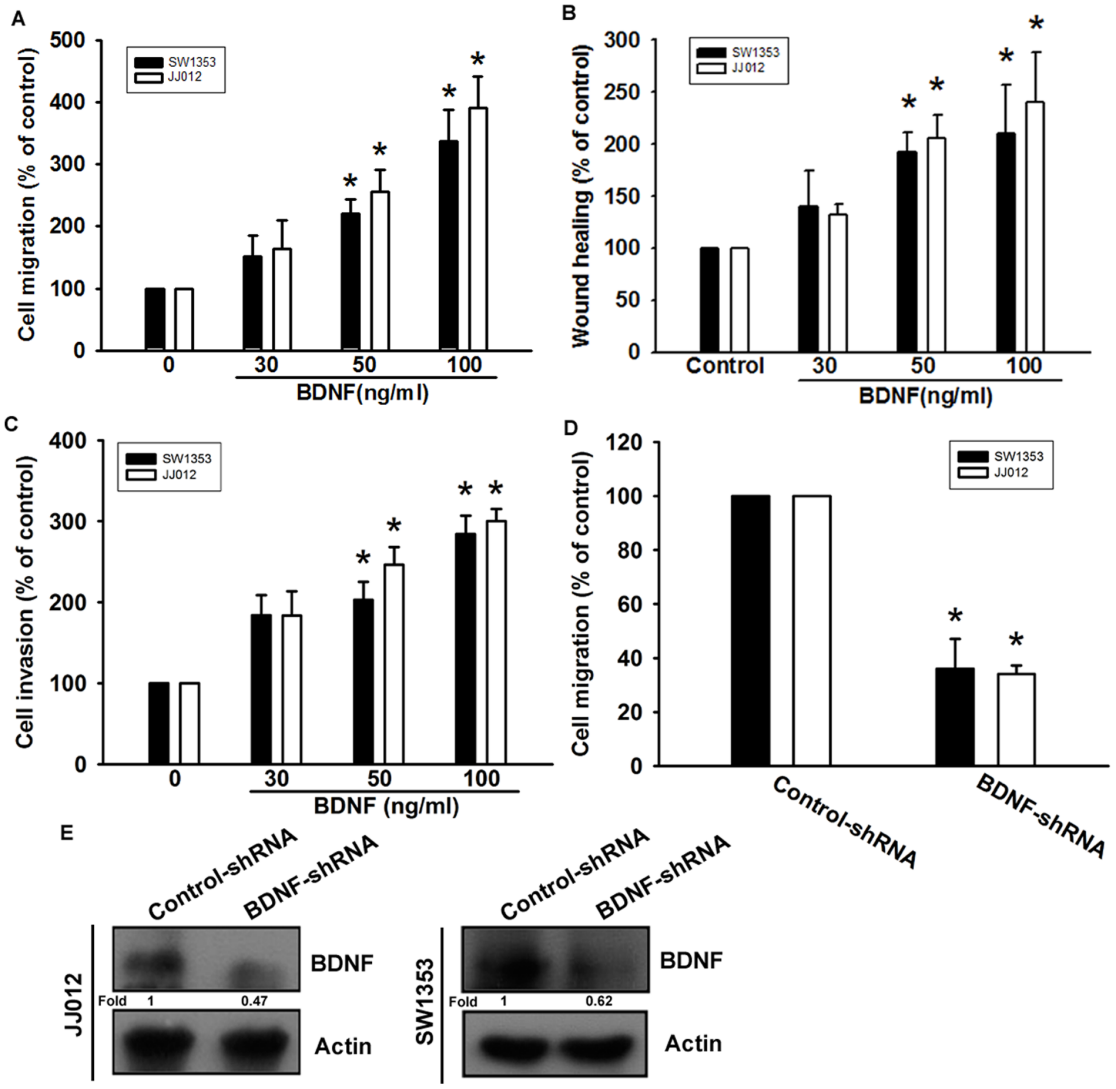
BDNF increased cell migration in chondrosarcoma cells. (A) Cells were added to the upper chamber, and then allowed to migrate for 24 h toward BDNF (30–100 ng/ml)-containing medium. (n = 4) (B) Cells were treated with BDNF for 24 h, and the wound-scratching assay was performed (n = 5). (C) Cells were added to the upper chamber, and then allowed to invasive for 24 h toward BDNF (30–100 ng/ml)-containing medium (n = 4). (D&E) The protein levels of BDNF and *in vitro* migratory activity of cells were measured using western blotting and a Transwell assay (n = 4). Results are expressed as the mean ± SEM. *, *p*<0.05 compared with the control group. ^#^, *p*<0.05 compared with the BDNF-treated group.

It has been reported that BDNF stimulates directional migration and invasion of human cancer cells [21], [22]. We next examined the migratory activity of human chondrosarcoma using the Transwell assay. Stimulation of human chondrosarcoma cells (JJ012 and SW1353 cells) with BDNF increased migratory activity in a dose-dependent manner (Fig. 2A). In addition, the wound-scratching assay demonstrated that BDNF increased wound healing activity in human chondrosarcoma cells (Fig. 2B). Treatment of chondrosarcoma cells with BDNF also increased their ability to invade a Matrigel basement membrane matrix (Fig. 2C). To confirm BDNF- mediated cell migration in human chondrosarcoma cells, cells expressing BDNF-shRNA were established. BDNF expression was reduced by BDNF-shRNA in JJ012/BDNF-shRNA and SW1353/BDNF-shRNA cells (Fig. 2E). However, knockdown of BDNF expression inhibited the migratory ability of JJ012 and SW1353 cells (Fig. 2D). Therefore, BDNF expression is associated with an invasive and metastatic phenotype in chondrosarcoma cells.

### Effect of β5 Integrin Up-regulation in BDNF-induced Motility of Chondrosarcoma Cells

**Figure 3:**
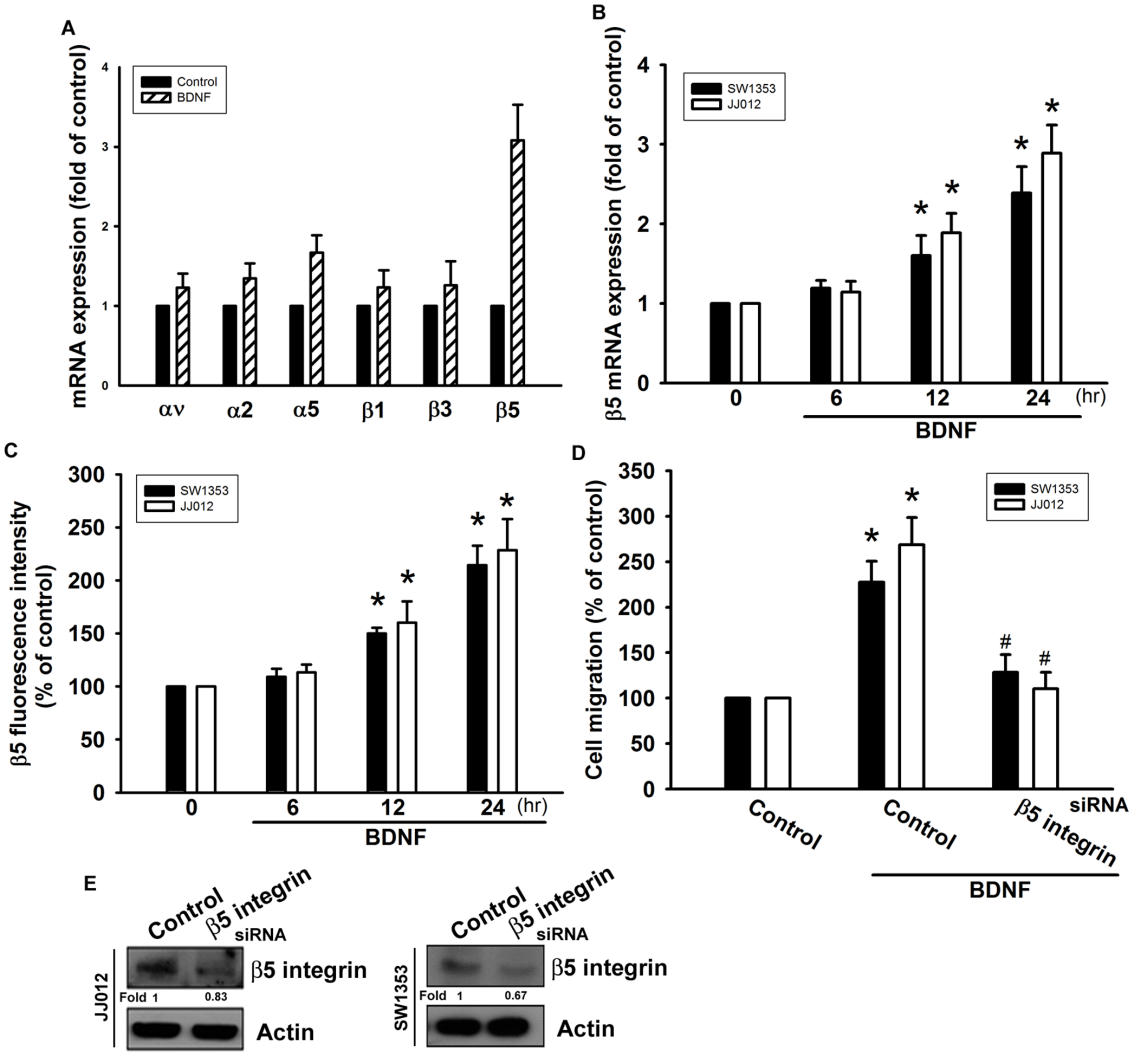
BDNF-directed migration activity of human chondrosarcoma cells involves up-regulation of β5 integrin. (A) JJ012 cells were incubated with BDNF (50 ng/ml) for 24 h, and mRNA expression of αv, α2, α5, β1, β3, and β5 integrin was examined by qPCR (n = 4). (B&C) Cells were incubated with BDNF (50 ng/ml) for the indicated time intervals, and mRNA and cell surface β5 integrin expression were examined by qPCR and flow cytometry (n = 5). (D&E) Cells were transfected with β5 siRNA for 24 h and then allowed to migrate for 24 h toward BDNF (50 ng/ml)-containing medium. The *in vitro* migration and β5 expression was measured with the Transwell assay and western blotting (n = 5). Results are expressed as the mean ± SEM. *, *p*<0.05 compared with the control. ^#^, *p*<0.05 compared with the BDNF-treated group.

Previous studies have shown that integrin activation mediates the migration and metastasis of human chondrosarcoma cells [30], [31]. We therefore hypothesized that integrins are involved in BDNF-induced chondrosarcoma cell migration. Incubation of cells with BDNF increased expression of β5 but not αv, α2, α5, β1, and β3 integrins (Fig. 3A). We further confirmed that treatment of chondrosarcoma cells with BDNF increased the mRNA and cell surface expression of β5 integrin in a time-dependent manner (Fig. 3B&C). To examine whether β5 integrin is involved in the BDNF-induced increase in cell migration, we transfected cells with siRNA against β5 integrin for 24 h. Western blot analysis showed that protein expression levels of β5 integrin were suppressed (Fig. 3E). In addition, transfection of cells with β5 integrin siRNA significantly abolished BDNF-mediated cell migration (Fig. 3D), suggesting that β5 integrin is crucial to mediating the function of BDNF in tumor cell migration.

### BDNF-promoted Chondrosarcoma Cell Migration via the TrkB Receptor

**Figure 4:**
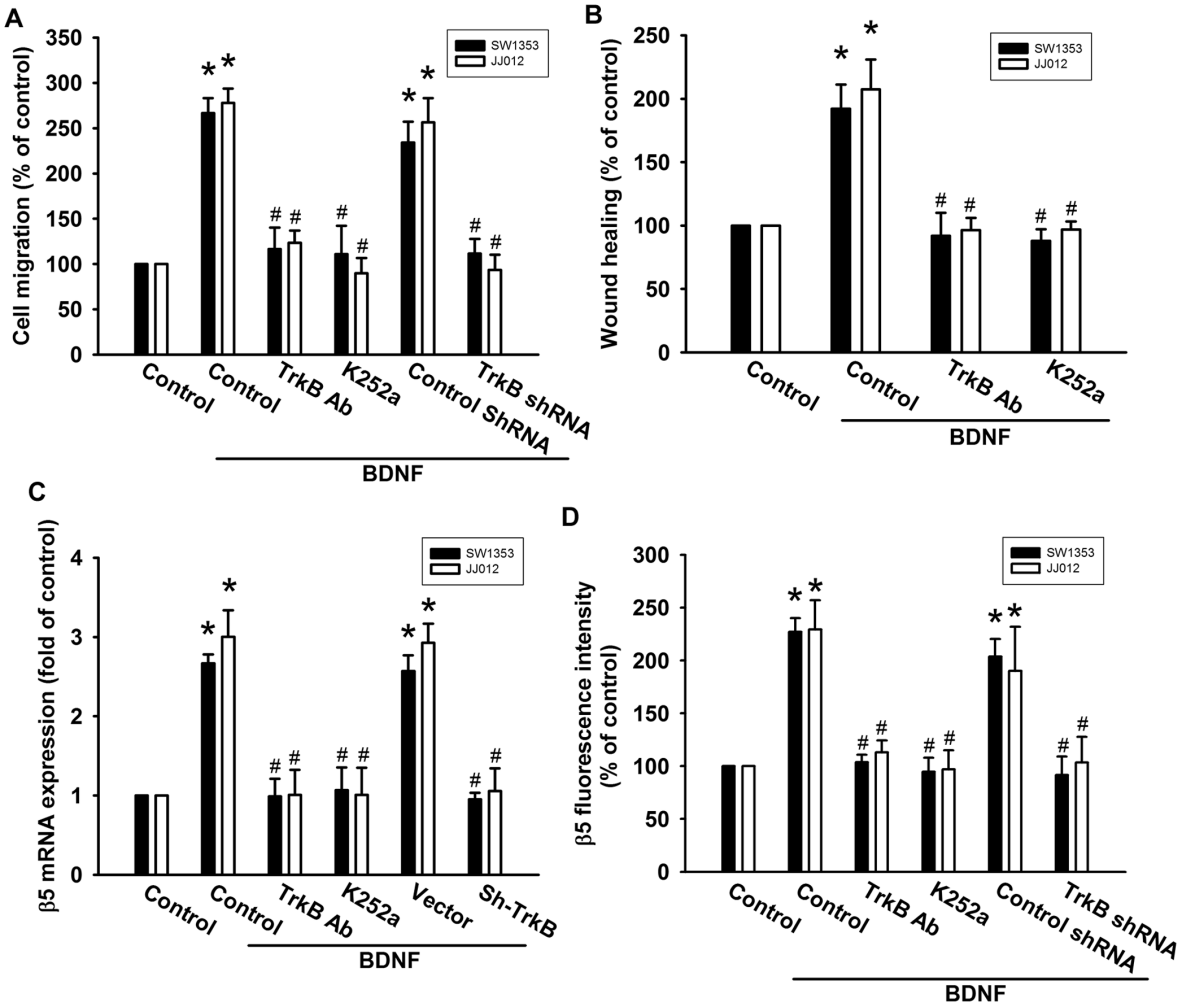
TrkB receptor is involved in BDNF-mediated migration of human chondrosarcoma cells. (A&B) Cells were pretreated with K252a (50 nM) or TrkB monoclonal antibodies (5 µg/ml) for 30 min or transfected with TrkB shRNA for 24 h and then allowed to migrate for 24 h toward BDNF (50 ng/ml)-containing medium. The *in vitro* migration and wound healing activity was measured (n = 5). (C&D) Cells were pretreated with K252a (50 nM) or TrkB mAb (5 µg/ml) for 30 min followed by stimulation with BDNF, and mRNA and cell-surface β5 integrin expression was examined using qPCR and flow cytometry (n = 4). Results are expressed as the mean ± SEM. *, *p*<0.05 compared with the control. ^#^, *p*<0.05 compared with the BDNF-treated group.

It has been reported that BDNF exerts its effects through activation of its specific receptor, TrkB [32], [33]. Therefore, we next determined whether the TrkB receptor is involved in BDNF-mediated cell migration in human chondrosarcoma cells. Pretreatment of cells with the TrkB receptor inhibitor K252a or TrkB receptor monoclonal antibody reduced BDNF-induced increases in cell migration (Fig. 4A&B). In addition, transfection of cells with TrkB shRNA also decreased BDNF-induced cell migration (Fig. 4A&B). Moreover, transfection of cells with TrkB shRNA or pretreatment of cells with the TrkB inhibitor and antibody effectively inhibited BDNF-increased β5 integrin expression of chondrosarcoma cells (Fig. 4C&D). These data suggest that the BDNF/TrkB interaction plays a critical role in integrin expression and migration in chondrosarcoma cells.

### PI3K and Akt Activation are Involved in BDNF-induced Cell Migration and β5 Integrin Expression in Chondrosarcoma Cells

**Figure 5:**
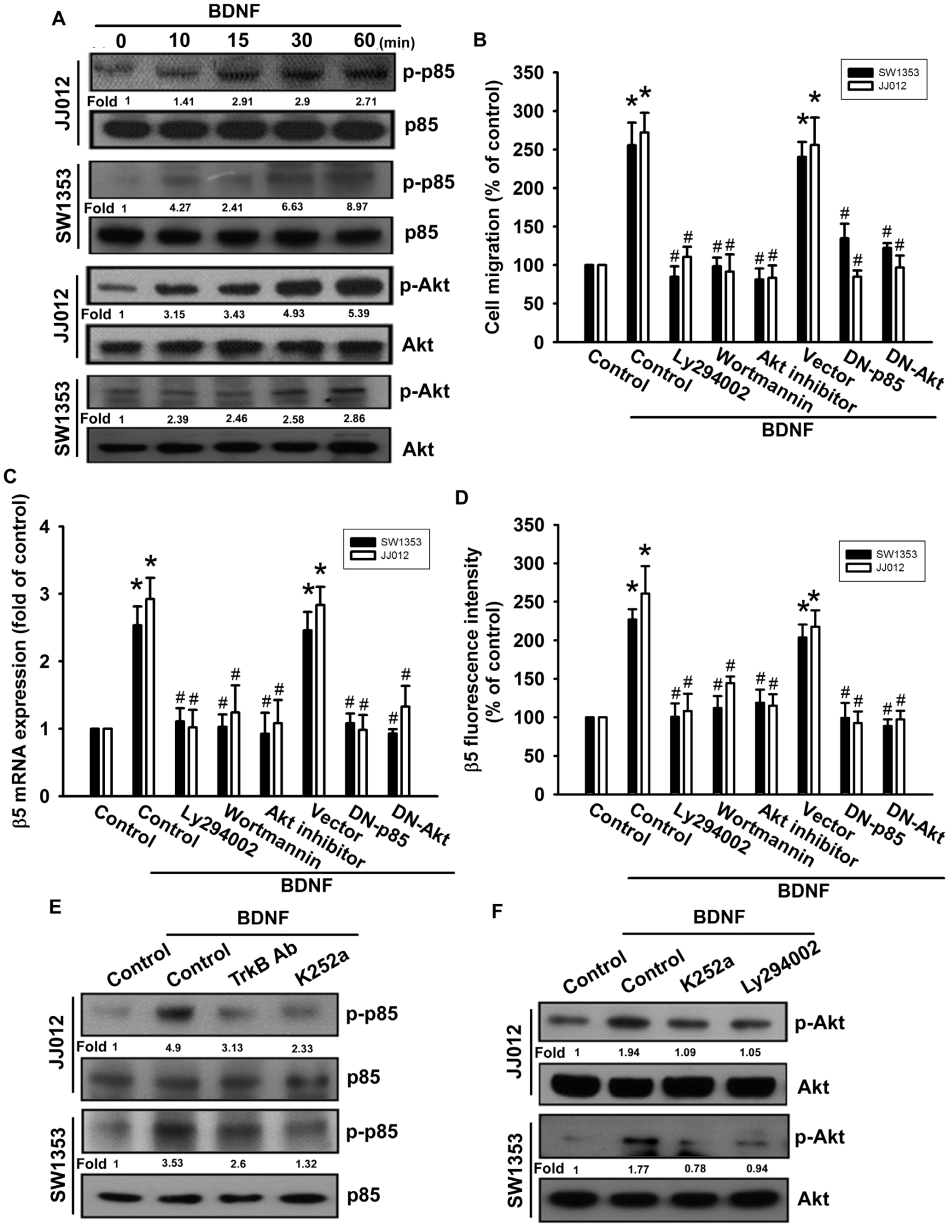
PI3K/Akt pathway is involved in BDNF-induced migration and β5 integrin up-regulation in human chondrosarcoma cells. (A) Cells were incubated with BDNF (50 ng/ml) for the indicated time intervals, and p-p85 and p-Akt was examined by western blotting (n = 5). (B) Cells were pretreated for 30 min with Ly294002 (10 µM), wortmannin (1 µM), and Akt inhibitor (1 µM), or transfected with dominant negative (DN) mutants of p85 and Akt for 24 h, and then allowed to migrate for 24 h toward BDNF (50 ng/ml)-containing medium. *In vitro* migration was examined using a Transwell assay (n = 4). (C&D) Cells were pretreated for 30 min with Ly294002 (10 µM), wortmannin (1 µM), and Akt inhibitor (1 µM), or transfected with dominant negative (DN) mutants of p85 and Akt for 24 h, followed by stimulation with BDNF. The mRNA and cell-surface β5 integrin expression was examined using qPCR and flow cytometry (n = 5). Cells were pretreated with K252a and TrkB mAb (E) or K252a and Ly294002 (F) for 30 min. This treatment was followed by stimulation with BDNF (50 ng/ml) for 15 min, and p85 (E) and Akt (F) phosphorylation were examined (n = 4). Results are expressed as the mean ± SEM. *, *p*<0.05 compared with the control. ^#^, *p*<0.05 compared with the BDNF-treated group.

PI3K/Akt is a common downstream molecule of the TrkB receptor [34]. Stimulation of cells with BDNF increased the phosphorylation of p85 (Fig. 5A). Pretreatment of chondrosarcomas with PI3K inhibitors (Ly294002 and wortmannin) abolished BDNF-mediated cell migration and β5 integrin expression (Fig. 5B–D). The PI3K-dependent signaling pathway is known to cause enzymatic activation of Akt Ser^473^ residue phosphorylation [35]. To explore the role of PI3K/Akt in cancer migration and integrin expression, we next investigated Akt Ser^473^ phosphorylation in response to BDNF treatment. As shown in Fig. 5A, stimulation of cells with BDNF led to significant induction of Akt Ser^473^ phosphorylation in a time-dependent manner. However, pretreatment with an Akt inhibitor greatly reduced BDNF-induced migration and integrin expression in chondrosarcoma cells (Fig. 5B–D). Furthermore, transfection of cells with p85 and Akt mutant also inhibited BDNF-induced migration and β5 integrin expression in chondrosarcoma cells (Fig. 5B–D). In contrast, BDNF-induced p85 and Akt phosphorylation was inhibited upon pretreatment of cells with TrkB antibody, K252a, and Ly294002 (Fig. 5E&F). From these results, it appears that the BDNF/TrkB axis acts through the PI3K/Akt-dependent signaling pathway to enhance β5 integrin expression and cell migration in human chondrosarcoma cells.

### NF-κB is Involved in BDNF-enhanced Migration and β5 Integrin Expression

**Figure 6:**
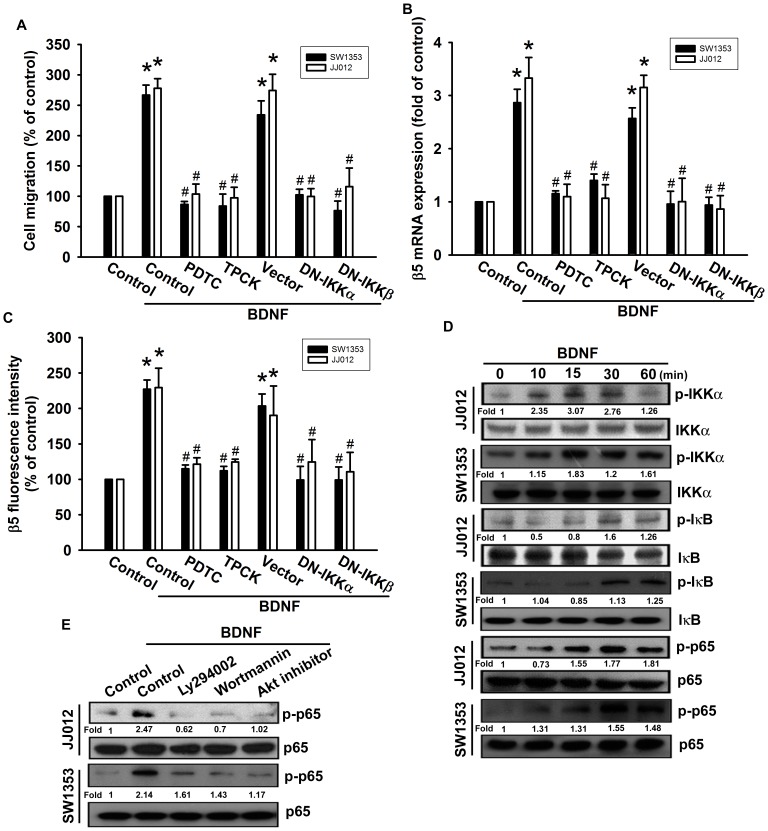
BDNF induces cell migration and β5 integrin up-regulation through NF-κB. (A) Cells were pretreated for 30 min with PDTC (10 µM) and TPCK (3 µM) or transfected with dominant negative (DN) mutants of IKKα or IKKβ for 24 h, and then allowed to migrate for 24 h toward BDNF (50 ng/ml)-containing medium. *In vitro* migration was examined using a Transwell assay (n = 5). (B&C) Cells were pretreated for 30 min with PDTC and TPCK or transfected with IKKα or IKKβ mutants for 24 h, followed by stimulation with BDNF. The mRNA and cell-surface β5 integrin expression was examined using qPCR and flow cytometry (n = 4). (D) Cells were incubated with BDNF for the indicated time intervals, and p-IKK, p-IκBα, and p-p65 was examined by western blotting (n = 5). (E) Cells were pretreated for 30 min with Ly294002, wortmannin, and Akt inhibitor, followed by stimulation with BDNF, and p-p65 expression was examined by western blotting (n = 4). Results are expressed as the mean ± SEM. *, *p*<0.05 compared with the control. ^#^, *p*<0.05 compared with the BDNF-treated group.

Recent studies have documented that NF-κB activation is necessary for the migration and invasion of human chondrosarcomas [36], [37]. To examine whether NF-κB activation is involved in the signal transduction pathway leading to migration and integrin expression caused by BDNF, we used the NF-κB inhibitor PDTC. PDTC inhibited the enhancement of migration and β5 integrin expression induced by BDNF (Fig. 6A–C). In addition, pretreatment of cells with an IκB protease inhibitor TPCK also antagonized the potentiating action of BDNF (Fig. 6A–C). We further examined the upstream molecules involved in BDNF-induced NF-κB activation. Transfection with IKKα or IKKβ mutants markedly inhibited BDNF-induced cell migration and β5 integrin expression (Fig. 6A–C). Furthermore, stimulation of chondrosarcoma cells with BDNF promoted IKKα/β, IκBα, and p65 phosphorylation in a time-dependent manner (Fig. 6D). Pretreatment of cells with Ly294002, wortmannin, or Akt inhibitor significantly reduced BDNF-induced p65 phosphorylation (Fig. 6E).

**Figure 7:**
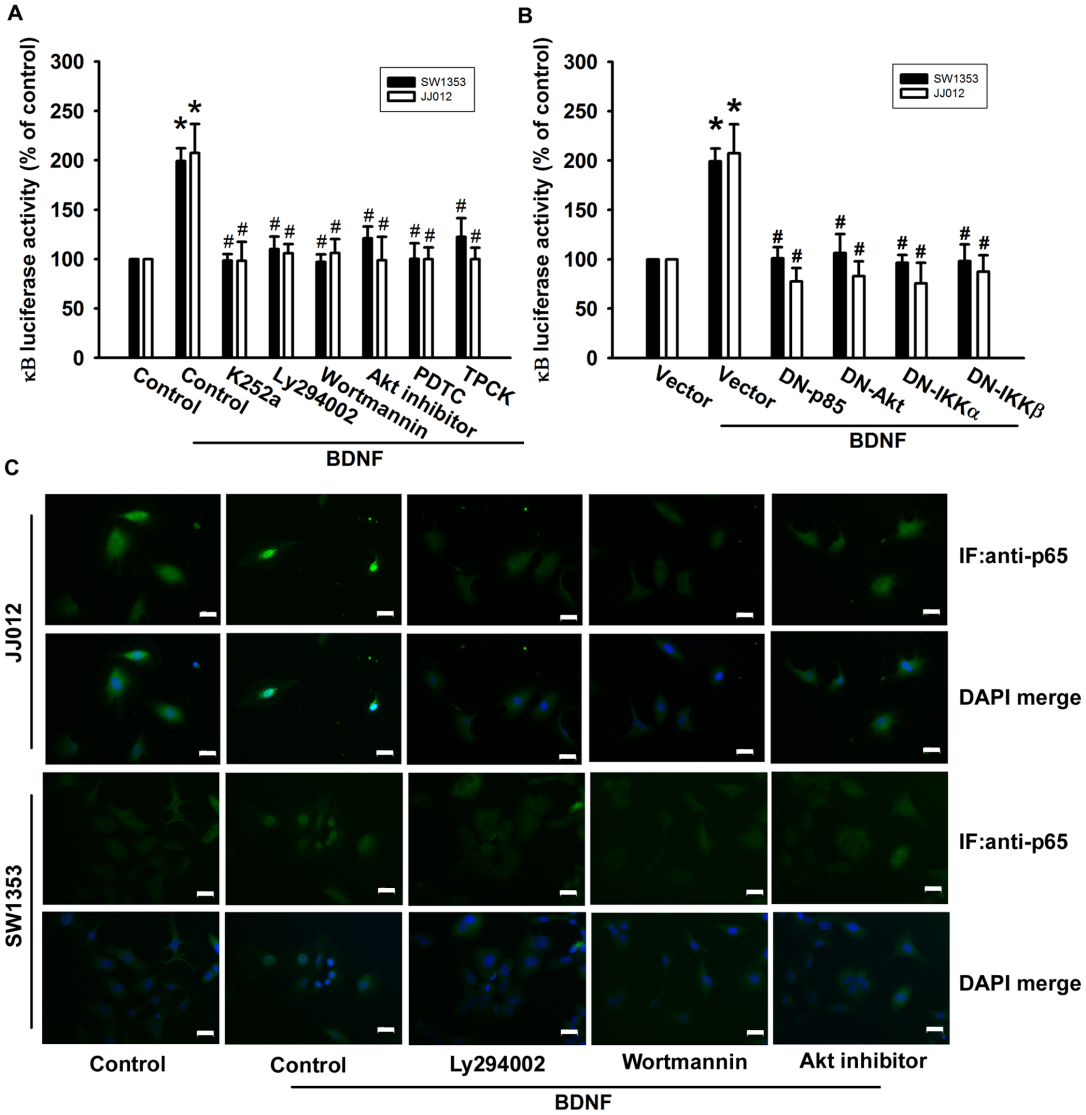
TrkB/PI3K/Akt pathway is involved in BDNF-mediated NF-κB activation. (A&B) Cells were pretreated with Ly294002, wortmannin, Akt inhibitor, PDTC, and TPCK for 30 min or transfected with mutants of p85, Akt, IKKα, and IKKβ before exposure to BDNF. NF-κB luciferase activity was measured, and the results were normalized to β-galactosidase activity and expressed as the mean ± SEM. for 3 independent experiments performed in triplicate (n = 5). (C) Cells were pretreated with Ly294002, wortmannin, and Akt inhibitor for 30 min then stimulated with BDNF for 60 min, and p65 immunofluorescence staining was examined (n = 4). Size bar = 20 µm. Results are expressed as the mean ± SEM. *, *p*<0.05 compared with the control. ^#^, *p*<0.05 compared with the BDNF-treated group.

To directly assess NF-κB activation after BDNF treatment, chondrosarcoma cells were transiently transfected with κB-luciferase as an indicator of NF-κB activation. BDNF treatment of chondrosarcoma cells for 24 h caused an increase in κB-luciferase activity (Fig. 7A). In addition, pretreatment of cells with K252a, Ly294002, wortmannin, Akt inhibitor, PDTC, or TPCK blocked BDNF-enhanced κB-luciferase activity (Fig. 7A). Moreover, the BDNF-induced increase in κB-luciferase activity was abolished by co-transfection of cells with p85, Akt, IKKα, or IKKβ mutant (Fig. 7B). Pretreatment of cells with Ly294002, wortmannin, or Akt inhibitor also reduced BDNF-induced accumulation of p65 into the nucleus (Fig. 7C). Taken together, these data suggest that activation of the TrkB receptor, PI3K, and Akt are required for BDNF-induced NF-κB activation in human chondrosarcoma cells.

## Discussion

Chondrosarcoma is a rare but deadly form of bone cancer and is the second most common type of bone cancer, accounting for nearly 26% of all bone cancers [38]. The metastatic potential of conventional chondrosarcomas correlates well with the histologic tumor grade. Because of the relatively indolent growth rates of many low- and moderate-grade chondrosarcomas, approximately 15% of all metastatic disease-related deaths are more than 5 years after initial diagnosis [39]. Therefore, it is important to develop effective adjuvant therapy to prevent chondrosarcoma metastasis. We hypothesized that BDNF would help direct the metastasis of chondrosarcoma cells. Using western blotting and immunohistochemical analysis, we found that expression of BDNF in human chondrosarcoma tissues was significantly higher than in primary chondrocytes and normal cartilage. Moreover, over-expression of BDNF shRNA inhibited the migratory ability of the cells. Therefore, BDNF expression is associated with a metastatic phenotype in chondrosarcoma cells. Exogenous BDNF also increased migration, wound healing, and invasion in human chondrosarcoma. One of the mechanisms underlying BDNF-directed migration was transcriptional up-regulation of β5 integrin and activation of TrkB receptor, PI3K, Akt, and NF-κB signaling pathways.

Integrins link the ECM to intracellular cytoskeletal structures and signaling molecules and are implicated in the regulation of a number of cellular processes, including adhesion, signaling, motility, survival, gene expression, growth, and differentiation [15]. In the present study, BDNF increased mRNA levels of only β5 but no other integrin. We also used β5 integrin siRNA to examine the role of β5 integrin and found that it inhibited BDNF-induced cancer cell migration. The experimental results indicate that BDNF promotes migratory ability through the up-regulation of β5 integrin in human chondrosarcoma cells. In addition to cancer metastasis, a similar signaling pathway has also been reported in breast cancer migration, which involved β5 integrin up-regulation [40] and activation, and mediated the growth and metastasis of murine mammary carcinomas [41], as well as in osteosarcoma migration, which involved β5 integrin activation [42]. Taken together, these results suggest that β5 integrin activation has an important role in treatment of cancer metastasis.

This key oncogenic signaling pathway has been linked to TrkB receptor activation, including up-regulation of the PI3K/Akt cascade [43]. We demonstrated that PI3K inhibitors and an Akt inhibitor could inhibit BDNF-induced migration and β5 integrin expression, suggesting that PI3K/Akt activation is a requisite event in BDNF-induced motility in these cells. This was confirmed by the observation that p85 and Akt mutants inhibited the enhancement of migration and β5 integrin expression in human chondrosarcoma cells. Incubation of cells with BDNF increased PI3K and Akt phosphorylation. Pretreatment of cells with K252a and Ly294002 reduced BDNF-mediated Akt phosphorylation. These data suggest that the PI3K-dependent Akt pathway is required for BDNF-induced β5 integrin expression and cancer migration. In contrast to PI3K/Akt signaling, ERK activation has been described a downstream molecule of β5 integrin [44]. We also found that ERK inhibitor PD98059 reduced BDNF-increased cell migration (data not shown). Stimulation of chondrosarcoma cells with BDNF enhanced ERK phosphorylation (data not shown). Therefore, ERK may play a role in BDNF-induced cell motility. Whether PI3K/Akt cross talk with ERK after BDNF simulation is needs further examination.

NF-κB has been shown to control the induction of transcription of β5 integrin in human cancer cells [45]. The results of this study show that NF-κB activation contributes to BDNF-induced migration and β5 integrin expression in human chondrosarcoma cells and that inhibitors of the NF-κB-dependent signaling pathway, including PDTC or TPCK, inhibit BDNF-induced β5 integrin expression and cancer migration. In an inactivated state, NF-κB is normally held in the cytoplasm by the inhibitor protein IκB. Upon stimulation, such as by tumor necrosis factor-α, IκB proteins become phosphorylated by the multi-subunit IKK complex, which targets IκB for ubiquitination, and are then degraded by the 26S proteasome. Finally, the free NF-κB is translocated to the nucleus, where it activates the responsive gene [46]. In the present study, using transient transfection with κB-luciferase as an indicator of NF-κB activity, we also found that BDNF induced an increase in NF-κB activity. In addition, K252a, Ly294002, wortmannin, Akt inhibitor, PDTC, and TPCK or p85, Akt, IKKα and IKKβ mutants reduced BDNF-increased NF-κB promoter activity. These results indicate that BDNF acts through the TrkB, PI3K, Akt, IKKα/β, and NF-κB pathway to induce cell migration and β5 integrin expression in human chondrosarcoma cells.

The rate-limiting step in metastasis, and a critical stage in cancer progression, is the acquisition of motility by a tumor cell. Lung metastasis is the major cause of mortality for patients with chondrosarcoma. Here, we have revealed critical new insights into BDNF function and its role in chondrosarcoma metastasis. BDNF expression is up-regulated in chondrosarcoma patients, and promotes cell migration. Although the mechanisms involved in BDNF-induced metastasis are not yet completely understood, our study results provide evidence that BDNF acts via the TrkB receptor, PI3K, Akt, and NF-κB signaling pathways. Our data demonstrate the importance of BDNF in the metastasis of chondrosarcoma, and suggest the use of BDNF as a novel therapeutic target for the clinical treatment of chondrosarcoma.

## Supporting Information

Figure S1 **The histogram results from FACS analysis.** Cells were pretreated for 30 min with TrkB Ab, K252a, Ly294002, wortmannin, and Akt inhibitor, or transfected with dominant negative (DN) mutants of p85, Akt, IKKα, IKKβ, and sh-TrkB for 24 h, followed by stimulation with BDNF, the cell-surface β5 integrin expression was examined using flow cytometry (n = 4).
(DOC)
