# Supplementary material for: β5 Integrin Up-Regulation in Brain-Derived Neurotrophic Factor Promotes Cell Motility in Human Chondrosarcoma
Source: PLoS One. 2013 Jul 9;8(7):e67990. doi: 10.1371/journal.pone.0067990 (PMC3706611; doi:10.1371/journal.pone.0067990)
Supplement: Figure S1 — The histogram results from FACS analysis. Cells were pretreated for 30 min with TrkB Ab, K252a, Ly294002, wortmannin, and Akt inhibitor, or transfected with dominant negative (DN) mutants of p85, Akt, IKKα, IKKβ, and sh-TrkB for 24 h, followed by stimulation with BDNF, the cell-surface β5 integrin expression was examined using flow cytometry (n = 4). (DOC) [file pone.0067990.s001.doc]

**Supplementary Figure**


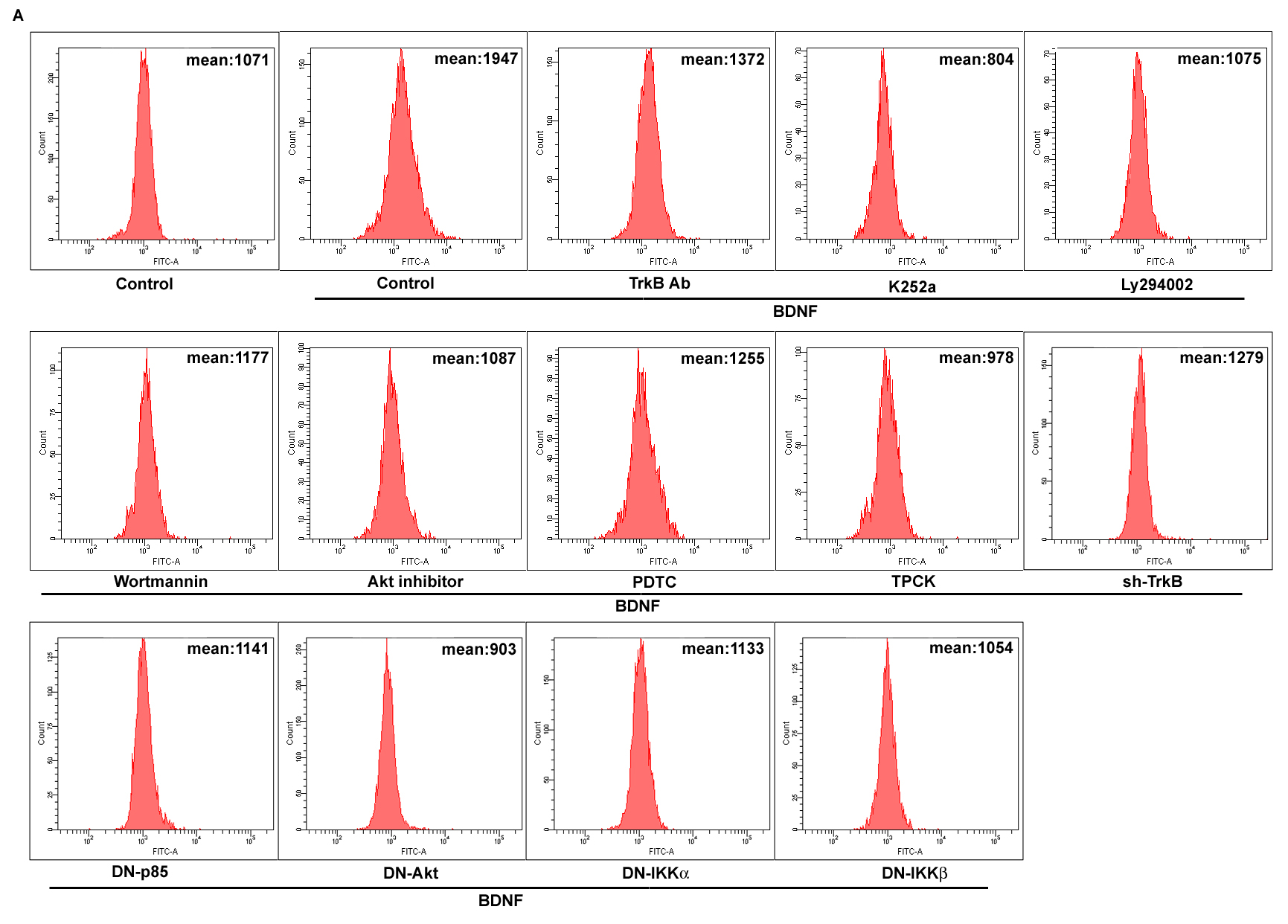


**Figure S1. The histogram results from FACS analysis**. Cells were pretreated for 30 min with TrkB Ab, K252a, Ly294002, wortmannin, and Akt inhibitor, or transfected with dominant negative (DN) mutants of p85, Akt, IKKα, IKKβ, and sh-TrkB for 24 h, followed by stimulation with BDNF, the cell-surface β5 integrin expression was examined using flow cytometry (n=4).
